# Epigenetic Regulation in Myocardial Fibroblasts and Its Impact on Cardiovascular Diseases

**DOI:** 10.3390/ph17101353

**Published:** 2024-10-10

**Authors:** Sumra Komal, Yuan Gao, Zhi-Mo Wang, Qing-Wen Yu, Pei Wang, Li-Rong Zhang, Sheng-Na Han

**Affiliations:** Department of Pharmacology, School of Basic Medical Sciences, Zhengzhou University, Zhengzhou 450001, China; sumra_komal1@outlook.com (S.K.); g15670764861@stu.zzu.edu.cn (Y.G.); wangzhimo@gs.zzu.edu.cn (Z.-M.W.); yqw101@gs.zzu.edu.cn (Q.-W.Y.); peiwang0707@zzu.edu.cn (P.W.); lrzhang@zzu.edu.cn (L.-R.Z.)

**Keywords:** cardiovascular diseases, cardiac fibroblast, epigenetic regulation, non-coding RNA, N6-methyladenosine

## Abstract

Myocardial fibroblasts play a crucial role in heart structure and function. In recent years, significant progress has been made in understanding the epigenetic regulation of myocardial fibroblasts, which is essential for cardiac development, homeostasis, and disease progression. In healthy hearts, cardiac fibroblasts (CFs) play a crucial role in synthesizing the extracellular matrix (ECM) when in a dormant state. However, under pathological and environmental stress, CFs transform into activated fibroblasts known as myofibroblasts. These myofibroblasts produce an excess of ECM, which promotes cardiac fibrosis. Although multiple molecular mechanisms are associated with CF activation and myocardial dysfunction, emerging evidence highlights the significant involvement of epigenetic regulation in this process. Epigenetics refers to the heritable changes in gene expression that occur without altering the DNA sequence. These mechanisms have emerged as key regulators of myocardial fibroblast function. This review focuses on recent advancements in the understanding of the role of epigenetic regulation and emphasizes the impact of epigenetic modifications on CF activation. Furthermore, we present perspectives and prospects for future research on epigenetic modifications and their implications for myocardial fibroblasts.

## 1. Introduction

Cardiovascular diseases (CVDs) are the foremost contributor to morbidity and mortality globally [1]. Myocardial fibrosis, marked by an excessive buildup of extracellular matrix (ECM) proteins, frequently develops in different organs and is strongly associated with various CVDs [2]. Cardiac fibrosis is a prevalent pathophysiological change [3,4]. The progressive accumulation of ECM in cardiac fibrosis is closely associated with the abnormal activation and proliferation of cardiac fibroblasts (CFs) [5,6]. These activated CFs, referred to as myofibroblasts, play a significant role in ECM production and contribute to cardiac fibrosis as primary effector cells [7]. In a healthy state, resident CFs maintain a relatively quiescent state, which is responsible for preserving the homeostasis of the ECM cardiac structure and aiding in electrophysiological conduction [8]. However, in the presence of cardiac injuries, such as myocardial infarction (MI) and ischemic injury, myofibroblasts increase ECM secretion, leading to myocardial fibrosis [9,10]. Despite numerous studies investigating CF activation, the precise molecular mechanisms underlying cardiac pathophysiology remain poorly understood. Extensive research demonstrates that epigenetic modifications significantly impact the activation of CFs and the expression of fibrosis-related genes [11,12,13].

Epigenetic regulation, which refers to heritable changes in gene expression without alterations in DNA sequences, has emerged as a key mechanism governing the functional plasticity of myocardial fibroblasts [14]. Epigenetic modifications, including DNA methylation and histone and RNA modifications, exert profound effects on gene expression patterns in various cell types [15]. Additionally, epigenetics serves as a bridge between genetics and environmental factors, providing an explanation for genetic phenomena that genetics alone cannot elucidate [16]. Epigenetic modifications are reversible and are facilitated by writers, such as DNA methyltransferases (DNMTs) and histone acetyltransferases (HATs), readers, including methyl-binding proteins (MBPs), and erasers, comprising DNA demethylases and histone deacetylases (HDACs) [17]. This reversibility offers opportunities for therapeutic interventions to correct aberrant gene expression by targeting these regulatory mechanisms rather than relying solely on genetic alterations [18]. Furthermore, various stimuli and stressors can induce changes in the epigenetic landscape of CFs, thereby influencing the expression of the genes involved in CVD progression [19]. Therefore, investigating the role of epigenetics in myocardial dysfunction may provide novel perspectives for the development of targeted therapies. This review aims to provide an overview of the current research progress on the epigenetic regulation of myocardial fibroblasts. Here, we discuss key epigenetic modifications implicated in myocardial fibroblast activation and their functional consequences. Furthermore, the signaling pathways and molecular mechanisms involved in orchestrating epigenetic changes in these cells highlight the potential therapeutic implications of targeting epigenetic regulators in the context of myocardial fibrosis.

## 2. Epigenetic Mechanisms Influencing the Regulation of Cardiac Fibroblasts

**Figure 1:**
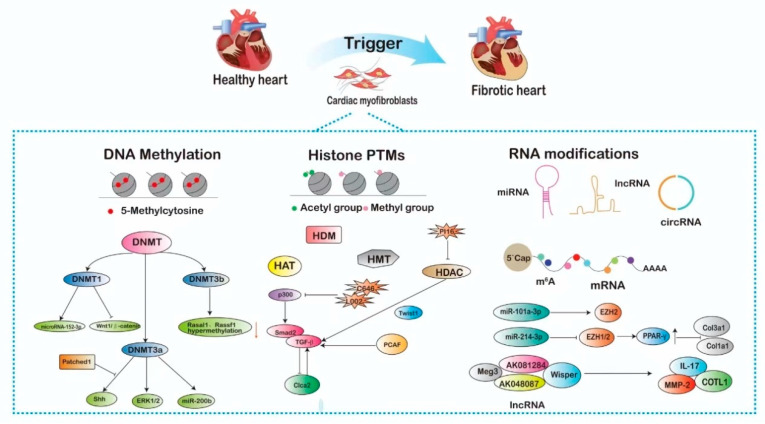
Epigenetic control of cardiac fibroblasts. An overview of the epigenetic mechanisms that play a pivotal role in regulating the behavior and function of cardiac fibroblasts (CFs). Epigenetic modifications, including DNA methylation, histone modifications, and non-coding RNAs, influence gene expression patterns in CFs, contributing to cardiac tissue remodeling and pathology. Understanding these epigenetic mechanisms is crucial for unraveling the complex biology of CFs and developing targeted therapies for heart-related conditions. DNMT, DNA methyltransferase; HAT, histone acetyltransferase; HDAC, histone deacetylase; HDM, histone demethylase; HMT, histone methyltransferases; m^6^A, N6-methyladenosine.

CFs represent the predominant non-myocytic cell type within the heart and play a pivotal role in preserving myocardial structure and function [20]. Under pathological conditions, such as MI or chronic pressure overload, CFs undergo phenotypic changes, become activated, and contribute to cardiac remodeling and fibrosis [21]. Recent investigations [22] have emphasized the role of epigenetic modifications in controlling CF activation and the subsequent development of fibrotic responses (Figure 1).

### 2.1. DNA Methylation-Mediated Activation of Cardiac Fibroblast

DNA methylation, a widely studied epigenetic modification in mammals, involves dynamic alterations in methylation patterns facilitated by two primary processes: de novo DNA methylation and demethylation. These processes are regulated by a cascade of enzymes serving as writers, readers, and erasers. In mammals, DNA methyltransferases (DNMTs) and demethylases work together to modulate the levels of DNA methylation; DNMTs facilitate the transfer of a methyl group from S-adenosyl-L-methionine (SAM) to the fifth carbon of the cytosine (C) base, resulting in the formation of 5-methylcytosine (5mC) [23,24]. In general, DNA methylation primarily occurs at cytosine–phosphate–guanine (CPG) dinucleotides, which are predominantly found in the promoter and exon regions of human genes [25].

DNA methylation mediated by DNMTs is a well-established epigenetic modification involved in regulating gene expression [24]. Studies have identified specific genes whose methylation status is altered during CF activation, including those involved in ECM synthesis, inflammation, and tissue remodeling. Furthermore, the pharmacological and genetic manipulation of DNMT activity has demonstrated the potential of targeting DNA methylation as a therapeutic strategy to modulate CFs.

In mammals, DNMTs, such as DNMT1, DNMT3a, and DNMT3b, function as writers, which have methyltransferase activity. DNMT3a and DNMT3b are involved in initiating new DNA methylation patterns, called de novo methylation, whereas DNMT1 is responsible for maintaining the existing DNA methylation state [25,26].

Recent studies have suggested that the inhibition of DNMT3a prevents hypoxia-induced CF activation and cardiac fibrosis [27]. In a rat model of isoprenaline (ISO)-triggered cardiac fibrosis, DNMT3a plays a crucial role in facilitating the activation of cardiac fibroblasts (CFs) and the progression of fibrosis through the ERK1/2 signaling pathway [28,29,30]. Additionally, Hedgehog signaling (Shh) is vital for the proliferation of CF, with Patched1 functioning as a negative regulator of this pathway [31]. Importantly, DNMT3a has been shown to inhibit the Patched1 signaling pathway via hypermethylation, which, in turn, promotes CF proliferation in models of cardiac fibrosis [32]. The pro-fibrotic effects associated with DNMT3a are also linked to the activation and proliferation of CF in an ISO-induced fibrosis model [33]. Furthermore, DNMT3a regulates autophagy in fibroblasts via miR-200b, suggesting a new therapeutic pathway for cardiac fibrosis [34]. Similarly, DNMT3b is involved in hypoxia-induced CF activation and the inhibition of fibrosis-related protein synthesis [35]. Methyl sequence analysis revealed abnormal methylation patterns in the promoters of Ras protein activator-like 1 (*Rasal1*) and Ras-related domain family 1 (*Rassf1*) in transverse aortic constriction (TAC)-induced cardiac fibrosis. Danhong injection (DHI), a traditional Chinese herbal remedy containing *Salvia miltiorrhiza* and *Carthamus tinctorius*, inhibits the hypermethylation of Rasal1 and Rassf1 by modulating DNMT3b expression in CFs. This therapeutic approach shows promise in mitigating cardiac fibrosis and enhancing cardiac function [36,37].

DNMT1 has also been implicated in fibrotic diseases [38,39]. In diabetic cardiac fibrosis, increased DNMT1 expression in CFs leads to the hypermethylation of the suppressor of cytokine signaling 3 (*SOCS3*) promoter, resulting in the silencing and promotion of CF activation [28]. Moreover, DNMT1-mediated methylation in ISO-induced cardiac fibrosis inhibits microRNA-152-3p, promoting CF activation and proliferation via the Wnt1/β-catenin signaling pathway [34]. Notably, DNA methylation does not occur in isolation but rather through cross-talk with other processes. During myocardial hypoxia, HIF-1α-induced reactive oxygen species (ROS) upregulate DNMT1 and DNMT3b expression, decrease Rassf1A expression through Snail activation, and lead to the increased synthesis of fibrosis markers [33]. In summary, DNA methylation plays a significant role in regulating CF activation and myocardial fibrosis [40]. DNA methylation modifiers, such as DNMTs or ten-eleven translocation (TET) proteins, have shown promise in attenuating or reversing cardiac fibrosis [12].

There are three types of TET enzymes: TET1, TET2, and TET3. They catalyze the sequential oxidation of 5-methylcytosine (5mC) to 5-hydroxymethylcytosine (5hmC), 5-formylcytosine (5fC), and 5-carboxylcytosine (5caC), respectively, as part of the process of DNA demethylation [41,42]. It has been reported that pregnant SD rats consuming an adult Western diet exhibit a heightened susceptibility to developing cardiac dysfunction, which can be partly linked to reduced levels of TET enzymes under unfavorable maternal conditions [43]. In a clonal hematopoietic mouse model, the administration of TET2 mutant bone marrow cells resulted in increased cardiac hypertrophy and myocardial fibrosis in the mice [44]. CF was aggravated after angiotensin-II (Ang-II) challenge in TET2 knockout mice [45]. Several in vivo and in vitro experiments have confirmed that TET3 has the ability to reverse the abnormal methylation patterns present in the RASAL1 promoter, consequently preventing the progression of myocardial fibrosis [46].

However, various DNMT isoforms display diverse mechanisms in different cardiac fibrosis models, rendering precise targeting challenging [38]. This regulatory process encompasses intricate signaling pathways and molecules that may potentially give rise to unintended side effects. Hence, further research is imperative to uncover novel mechanisms of DNA methylation regulation in cardiac fibrosis and to identify emerging regulatory pathways to advance innovative approaches for diagnosis and treatment (Table 1).

### 2.2. Histone Post-Translational Modifications-Mediated Activation of Cardiac Fibroblast

Histones are highly conserved basic proteins found in the nucleus that perform essential functions in preserving the integrity of DNA structure, protecting genetic information, and modulating gene expression [47]. The fundamental unit of chromatin comprises two sets of four core histones (H2A, H2B, H3, and H4), which assemble into a histone octamer capable of wrapping approximately 145 base pairs of DNA [48]. Epigenetic regulation involves histone modifications, including various post-translational modifications (PTMs). These modifications take place at the NH2-terminal tails of histones, which extend from the nucleosome. They encompass various processes, including acetylation, methylation, phosphorylation, and ubiquitination [17]. They modify the architecture of chromatin and influence gene expression, in addition to the interaction between histones and other proteins [49]. Studies have shown that various histone-modifying enzymes and complexes have been implicated in CF activation, contributing to the transcriptional regulation of key genes involved in fibrosis [50]. Histone modifications through small-molecule inhibitors or genetic approaches have shown promise for attenuating CF activation and improving myocardial function [51].

#### 2.2.1. Histone Acetylation and Deacetylation in CFs

Modifications involving histone acetylation primarily occur at the amino-terminal sites of lysine residues, and this process is dynamic and reversible. These modifications are regulated by two groups of proteins: histone acetyltransferases (HATs) and histone deacetylases (HDACs) [40,47,52]. HATs and HDACs play crucial roles in modifying chromatin structure by adding or removing acetyl groups at specific sites. This modification controls gene expression by regulating gene accessibility to the factors [53]. HATs mediate the addition of acetyl groups, leading to the loosening of the chromatin structure. This allows transcription factors to bind more easily to genes, leading to enhanced gene transcription [54]. In contrast, HDACs remove acetyl groups, causing histones to bind tightly to genes. This inhibits the binding of transcription factors to the promoters, resulting in the suppression of gene transcription and expression [53,55]. Overall, HATs and HDACs work together to maintain the proper balance between chromatin structure and gene expression regulation. CF activation and excessive ECM deposition in the heart are closely linked to the epigenetic changes caused by histone acetylation [56]. In a model of cardiac fibrosis induced by high glucose levels, the activation of p300 was activated and facilitated fibrosis by engaging mothers against decapentaplegic homolog 2 (*Smad2*) pathways [57,58]. The acetyltransferase activity of p300 is also responsible for the epigenetic regulation of type I collagen, which interacts with Smad3 to induce fibrosis, and the pro-fibrotic cytokine TGF-β relies on p300 for collagen synthesis in fibroblasts [58,59,76]. The inhibition of p300 using compounds such as L002 and C646 effectively reverses hypertension-induced cardiac hypertrophy and fibrosis in vivo [77]. Additionally, curcumin, a natural inhibitor of p300-histone acetyltransferase, has been shown to attenuate the progression of heart failure (HF) in vivo [78]. Furthermore, the curcumin analog GO-Y030 is a specific inhibitor with potential therapeutic applications against fibrotic diseases. Furthermore, p300 is a potential target for anti-fibrotic treatment and is closely associated with CF activation [79]. In addition, another histone acetyltransferase, PCAF, is essential for the activation of cardiac fibroblasts in response to TGF-β1 [80]. Intriguingly, depletion of miR-134-5p has been found to protect against myocardial remodeling and cardiac fibrosis in a rat model of MI through the elevation of lysine acetyltransferase 7 expression and the acetylation of histone H3K14 [81].

Histone deacetylases (HDACs) are enzymes responsible for catalyzing the removal of acetyl groups from lysine residues, whether located on histone or non-histone proteins [82]. They are often referred to as “erasers” because they are responsible for reversing specific post-translational modifications (PTMs) on histones. HDACs are categorized into four distinct classes: I, II, III, and IV. Classes I, II, and IV HDACs operate through a zinc-dependent mechanism to promote the process of deacetylation, while class III HDACs rely predominantly on nicotinamide adenine dinucleotide (NAD+) for their activity [83]. Notably, class I HDACs, which encompass HDAC1, HDAC2, HDAC3, and HDAC8, have emerged as significant regulators of cardiac fibrosis through the activation of CFs, as supported by findings from both in vitro and in vivo studies [60,84,85]. Further investigations have revealed that HDAC1 is recruited to the promoter of Chloride channel accessory 2 (*Clca2)* by TGF-β through the involvement of Twist1. This recruitment leads to the elimination of histone H3/H4 acetylation, which inhibits Clca2 transcription. The downregulation of Clca2 facilitates the activation of CFs into myofibroblasts, contributing to the development of cardiac fibrosis. Conversely, the upregulation of Clca2 counteracts this process and mitigates cardiac fibrosis induced by TGF-β-induced cardiac fibrosis [61]. In addition to CF activation, HDAC1 promotes CF proliferation and migration [62]. Gene expression profiling revealed that peptidase inhibitor 16 (PI16) attenuated Ang-II-induced cardiac fibrosis by suppressing the expression of HDAC1 [86]. Mechanistically, mitigated Ang-II-induced levels of HDAC1 by modulating the HDAC1/p53 signaling pathway. This reduction in HDAC1 levels increases the acetylation of H3K18 and H3K27, which, in turn, inhibits CF proliferation and the expression of fibrosis-related genes. Thus, PI16 has been shown to markedly mitigate Ang-II-induced cardiac fibrosis [87]. In murine models subjected to pressure overload, MI, and ISO-induced cardiac injury, fibroblast proliferation exhibited an initial surge during the first week following the onset of cardiac remodeling. Subsequently, this proliferation returned to normal levels as inflammation subsided [88]. Class I HDAC inhibitors, such as mocetinostat (MGCD0103), have shown significant suppression of CF proliferation and mitigation of fibrotic remodeling after cardiac injury [84]. The ability of HDAC inhibitors to reduce cardiac fibrosis is partially attributed to their capacity to inhibit the expansion of fibroblasts responsible for ECM production in the heart [89]. Furthermore, HDAC1 inhibition attenuated Ang-II-induced fibrosis by reducing mitochondrial oxidative stress. However, further studies are needed to fully understand the role of HDAC1 in cardiac fibrosis and explore potential therapeutic strategies targeting HDACs for the treatment of this condition [90]. Additionally, HDAC2, HDAC3, and HDAC8 have also been implicated in the context of cardiac fibrosis. HDAC2, in particular, may potentially have a significant effect on the progression of cardiac fibrosis [64,65,91].

#### 2.2.2. Histone Methylation and Demethylation in CFs

Histone methylation predominantly occurs at lysine (K) or arginine (R) residues within histone H3 and H4 tails [92]. The precise location and extent of methylation determine the transcriptional activity of the associated genes [93]. Histone methylation plays a crucial role in regulating chromatin condensation, leading to either an open state that facilitates chromatin transcription or a closed state linked with reduced transcription [70,94]. For instance, histone 3 lysine 4 triamcinolone (H3K4me3) is associated with promoter activity, whereas H3K9me3 trimethylation is commonly associated with transcriptional repression [95]. Catalytic enzymes responsible for histone methylation can be categorized into two groups: protein arginine methyltransferases (PRMTs) and protein lysine methyltransferases (KMTs). Conversely, histone lysine demethylase (KDM) removes histone methylation [96]. Furthermore, KMTs and KDMs have been identified as key regulators in the modulation of cardiac fibrosis. Moreover, research has indicated that the enhancer of zeste homolog 2 (*EZH2*) enhances vulnerability to fibrosis during pathological processes [67]. Interestingly, *EZH2* inhibition shows promise in reversing the fibrotic characteristics associated with cardiac diseases [97]. Notably, both the *EZH2* inhibitor, GSK126, and molecular silencing of *EZH2* have demonstrated effectiveness in inhibiting Angiotensin II (Ang-II)-induced activation, migration, and ECM production in atrial fibroblasts in animal models [98]. Additionally, these interventions have shown the potential to mitigate atrial enlargement and fibrosis caused by Ang-II, ultimately reducing vulnerability to atrial fibrillation (AF) [66].

miR-101a-3p has shown promise in preventing the onset of AF in rats by targeting EZH2, thereby inhibiting collagen synthesis and atrial fibrosis [68]. Similarly, miR-214-3p binds to EZH1/2, suppressing its transcriptional expression. This upregulated the expression of peroxisome proliferator-activated receptor-gamma (*PPAR-γ*) and inhibited the expression of Col1a1 and Col3a1 in myofibroblasts [99]. The long non-coding RNA (lncRNA) ANRIL plays a significant role in cardiac fibrosis in diabetic mice by influencing the expression of fibronectin (FN), vascular endothelial growth factor (*VEGF*), and type IV collagen (Col1α4). ANRIL interacts with multiple epigenetic regulators, including p300 and *EZH2* [69]. Moreover, lncRNA NEAT1 recruits *EZH2* to the promoter region of Smad7, leading to a reduction in *Smad7* expression. This ultimately contributes to the progression of cardiac fibrosis. Conversely, silencing NEAT1 has been found to significantly improve cardiac fibrosis and dysfunction induced by TAC in mice [100].

Histone lysine demethylases (KDMs) play pivotal roles in transcription reprogramming. Currently, two subfamilies of KDMs have been recognized: the JMJC domain-containing family (JMJD) and lysine-specific demethylases (LSD) [74,75]. The LSD family of demethylases specifically targets mono- and dimethylated histones H3K9 and H3K4, whereas the JMJD family of demethylases is capable of removing three methylation modifications. Notably, LSD1/KDM1 has emerged as a pivotal player in cardiac fibrosis as it directly acts on its substrates H3K4me1/2 and H3K9me1/2, thereby exerting an influence on cardiac remodeling [72,101]. Studies have shown that the inducible knockout of LSD1, specifically in myofibroblasts, effectively attenuates fibrosis, cardiac hypertrophy, and pressure overload-induced HF by suppressing the TGF-β signaling pathway [73]. Another KDM, KDM3A, is involved in the removal of H3K9me2/3. H3K9 methylation is downregulated in failing and hypertrophic hearts in both humans and mice [102]. In TAC-induced cardiac fibrosis, KDM3A binds to the promoter of tissue metalloproteinase inhibitor 1 (Timp1) and removes methylation modifications, leading to the activation of TIMP1 transcription. This, in turn, triggers CFs and promotes the progression of cardiac fibrosis [103].

TIMP1 has been recognized as a potential target of the pro-fibrotic effects of KDM3C. In a mouse model induced by Ang-II, KDM3C demethylates H3K9me2 on the Timp1 promoter, ultimately enhancing Timp1 transcriptional activation and consequently contributing to both cardiac fibrosis and CF activation [104]. It is essential to highlight that additional research is essential to comprehensively elucidate the specific mechanisms through which KDMs influence cardiac fibrosis, along with the potential therapeutic implications of targeting these demethylases. However, these findings provide valuable insights into the role of KDMs in cardiac fibrosis and may contribute to future therapeutic strategies aimed at mitigating fibrotic remodeling in the heart [22]. Furthermore, in spontaneously hypertensive rats (SHRs), MALAT1 recruits SUV39H1, resulting in the modification of histone H3 at lysine 9 (H3K9me3) at MyoD-binding loci. This molecular interaction contributes to the development of cardiac fibrosis [71,105]. Similarly, the non-SET domain methyltransferase disruptor of telomeric silencing 1-like (DOT1L) enhances the expression of splenic tyrosine kinase (SYK) by augmenting histone H3 modification at lysine 79 (H3K79me2) in the SYK promoter [106]. Elevated SYK levels subsequently activate the TGF-β1/Smad3 signaling pathway, contributing to CF proliferation and cardiac fibrosis. Notably, DOT1L led to a significant decrease in both cardiac injury and fibrosis associated with MI (Table 1) [107].

### 2.3. Non-Coding RNA-Mediated Activation of Cardiac Fibroblasts

Non-coding RNAs (ncRNAs) are crucial participants in a wide array of biological processes [108]. ncRNAs include microRNAs (miRNAs), long non-coding RNAs (lncRNAs), and circular RNAs (circRNAs), which are RNA transcripts that do not encode proteins but possess biological functions. These ncRNAs regulate post-transcriptional gene expression regulation [109].

#### 2.3.1. miRNAs in CFs

miRNAs, which are short ncRNAs less than 200 nucleotides in length, exert their biological functions by binding to the 3′ or 5′ untranslated regions (UTRs) of target mRNA molecules, leading to the inhibition of protein translation or degradation of mRNA, thereby achieving post-transcriptional gene silencing [110]. For example, miR-369-5p promotes fibroblast proliferation by modulating DNMT3A methylation and inhibiting the Patched1 pathway [36]. Likewise, miR-489 mitigates cardiac fibrosis through the regulation of HDAC2, which subsequently inhibits the activation and proliferation of fibroblasts [111]. Notably, miRNAs serve a dual role as upstream regulators of DNA methylation and histone modifications, and as direct influencers of fibrosis as downstream effectors [112]. Several miRNAs, including miR-23b, miR-27b, miR-125b, miR-99b-3p, miR-143-3p, miR-21, miR-27b-3p, and miR-9-5p, exert control over the activation, proliferation, and migration of CFs by targeting downstream fibrosis-related genes, like *Apelin*, *FBW7*, *Fgf1*, *FSL1*, *GSK-3β*, *p53*, *PTEN*, *SPRY3*, *Spry1/2*, and *TGIF1*, thereby promoting the development of fibrosis [113,114,115,116,117]. Several miRNAs, including miR-221/222, miR-1954, miR-590-3p, miR-15a/b, miR-15a/b, miR-21, and miR-29, inhibit fibrosis development [118,119,120]. The typical miRNAs associated with myocardial fibrosis and their respective functions are summarized as follows [121,122,123,124,125,126,127,128,129,130,131,132,133,134,135] (Table 2).

#### 2.3.2. LncRNAs in CFs

Long non-coding RNAs (lncRNAs), exceeding 200 nucleotides in length, are pivotal players in the development of cardiac fibrosis, with the ability to influence gene expression through direct interactions with DNA, RNA, and proteins [136]. Numerous lncRNAs are crucial regulators of the progression of cardiac fibrosis. For example, lncRNAs, such as AK081284, Meg3, Wisper, and AK048087, have been identified as promoters of CF activation and proliferation, achieved by modulating fibrosis-related genes, including *IL-17*, *MMP-2*, and *COTL1* [138,145,146,153]. Furthermore, lncRNA AK137033 plays a crucial role in modulating the stability of *Sfrp2* mRNA, while lncRNA PCFL is known to interact with miR-378 [137]. Collectively, these factors contribute to the regulation of cardiac fibrosis after MI. LncRNA Neat1, regulated by transcription factors p53 and HIF-1α, is elevated and delivered to CFs via extracellular vesicles (EVs), consequently initiating the expression of fibrosis-related genes [142]. lncRNA MIAT, an MI-related transcript, promotes myocardial fibrosis by competitively targeting miR-24, thereby promoting collagen expression [154]. Increased lncRNA PFL effectively interacts with the cardioprotective microRNA let-7d in CFs, inhibiting the interaction between the platelet-activating factor receptor and let-7d [155,156].

Wisper directly interacts with TIAR, an RNA-splicing protein, facilitating the interaction between TIAR and procollagen-lysine 2-oxoglutarate-5-dioxygenase 2 (Plod2). This interaction enhances the expression of collagen [145]. MIAT promotes myocardial fibrosis by competitively targeting miR-24, thereby promoting collagen expression [154]. LncRNA Kcnq1ot1 is upregulated in high glucose-induced myocardial fibrosis, which mediates the expression of caspase-1 by sponging miR-214-3p, thereby activating the *TGF-β1/Smads* signaling pathway, which ultimately leads to pyroptosis [144].

In contrast, certain lncRNAs exert inhibitory effects on cardiac fibrosis. The overexpression of lncRNA GAS5 can reduce the levels of *α-SMA* and *Col1A1*, which are markers of fibrosis, in CFs by inhibiting miR-21 expression, thereby alleviating cardiac fibrosis [143]. Crnde acts as a suppressor of CF activation, alleviating cardiac fibrosis associated with dilated cardiomyopathy (DCM) through a negative feedback mechanism that regulates *Smad3* transcriptional activation [139]. Additionally, Dioscin, a glucoside saponin derived from *Dioscorea nipponica* Makino, mitigates MI-induced cardiac fibrosis by elevating the levels of the lncRNA MANTIS. This, in turn, enhances the expression of genes associated with angiogenesis, including *SOX18*, *SMAD6*, *and COUP-TFII* [140]. Intriguingly, the histone demethylase JARID1B is also involved in the regulation of lncRNA MANTIS and the control of angiogenesis-related gene expression, including *SMAD6* [137,138,139,140,141,142,143,144,145,146,153] (Table 2).

#### 2.3.3. CircRNAs in CFs

It was shown that circRNA_0036176 could bind miR-218-5p and thus inhibit the development of myocardial fibrosis [157]. Another study showed that circ_0120051 inhibited the fibrotic phenotype of cardiac fibroblasts through the miR-144-3p/IDH2 axis. The expression of circ_0120051 was found to be significantly increased in the myocardium of heart failure patients and localized mainly in the cytoplasm of cardiomyocytes. The overexpression of circ_0120051 in mouse cardiac fibroblasts significantly inhibited the expression of fibrosis-related genes and the migration ability of the cells. Further studies confirmed that circ_0120051 exerted its inhibitory effect by specifically binding to miR-144-3p and increasing the expression of its target gene IDH2 [158].

### 2.4. RNA Modifications-Mediated Activation of Cardiac Fibroblast

RNA modifications directly influence its biological functions, with N6-methylated adenosine (m^6^A) emerging as the most prevalent epigenetic modification in eukaryotic RNA. Several RNAs have m^6^A marker sites, including mRNA, tRNA, rRNA, snRNAs, and lncRNA. This modification is crucial to the pathophysiology of cardiac fibrosis as it regulates the transport, degradation, and translation of RNA. The regulation of m^6^A modification involves three categories of effector proteins: m^6^A methyltransferase, including methyltransferase-like protein (METTL 3/5/14/16), and its removal requires the execution of demethylase fat mass obesity-associated protein (FTO) and AlkB homolog5 (*ALKBH5*), and m^6^A reader proteins equipped with the YTH structural domain (YTHDF1/2/3 and YTHDC1/2) are responsible for identifying specific m6A modification sites, which cause variable gene regulation outcomes [159].

The augmentation of m^6^A modification levels through METTL3 overexpression enhances collagen synthesis and promotes cardiac fibrosis [160]. Notably, genes associated with fibrosis that are modified by m6A and mediated by METTL3 play a significant role in the regulation of MetBil in the context of cardiac fibrosis following MI [161]. Another investigation revealed that the silencing METTL3 significantly reduced the expression levels of IGFBP3, leading to the inhibition of fibroblast activation and a decrease in cardiac fibrosis, both in vitro and in vivo. This finding proposes that METTL3 might play a crucial role in regulating the expression of *IGFBP3* and the activation of CFs through RNA epigenetic modifications [147]. WTAP promotes the oxidation of mitochondrial lipids as well as the proliferation and migration of fibroblasts, thereby contributing to the development of diabetic cardiac fibrosis. Mechanistically, the m6A methylation of the androgen receptor, mediated by WTAP, leads to its degradation, a process that relies on the involvement of YTHDF2. WTAP promotes mitochondrial lipid oxidation and fibroblast proliferation and migration to induce diabetic cardiac fibrosis. Mechanistically, WTAP-mediated m^6^A methylation of the androgen receptor induces its degradation, which is dependent on YTHDF2 [148].

Conversely, the expression of the m^6^A demethylase FTO is reduced in cardiac fibrosis induced by conditions, such as MI, hypoxia, and HF [149]. FTO overexpression inhibits CF activation, proliferation, and migration, thereby ameliorating cardiac fibrosis. FTO knockdown promoted the migration of CFs, increased the protein levels of Col-3, α-SMA and Col-1 in Ang II and LE-stimulated CFs, and enhanced the fluorescence intensity of α-SMA [150]. *ALKBH5* plays a pivotal role in the healing process following MI by enhancing the stability of ErbB4 mRNA through an m6A-dependent mechanism. Additionally, it facilitates the transformation of fibroblasts into myofibroblasts under hypoxic conditions, providing protection against cardiac rupture post-MI [151] (Table 1).

However, the study of m^6^A methylation in myocardial fibers is limited and is an area that requires urgent exploration.

## 3. Epigenetic Therapies and Cardiovascular Diseases

Epigenetic drugs are therapeutic agents engineered to reshape the epigenetic landscape of cells, influencing gene expression without changing the DNA sequence [15]. These drugs target critical epigenetic mechanisms, such as DNA methylation, histone modifications, and non-coding RNA activity, to reverse or alter pathological gene expression patterns linked to diseases like cancer, neurological disorders, and cardiovascular diseases (CVDs).

### 3.1. DNA Methylation Inhibitors

DNA methyltransferase inhibitors have potential in treating CVDs like coronary heart disease and HF by regulating gene methylation and expression levels. Studies have demonstrated that 5-Aza-dC (*decitabine*) can reduce atherosclerosis in Ldlr^−/−^ mice by inhibiting macrophage migration and adhesion to epithelial cells, decreasing macrophage infiltration into atherosclerotic plaques, and lowering inflammatory gene expression [152]. The DNMT inhibitor RG108 is instrumental in addressing atherosclerosis and coronary heart disease through its ability to inhibit the activities of DNMT1 and DNMT3a. Furthermore, RG108 contributes to the mitigation of myocardial fibrosis and hypertrophy by effectively hindering DNA methyltransferase activity [162,163].

Studies indicate that the DNA methylation inhibitor 5-azacytidine can mitigate the negative effects of tumor necrosis factor-α on SECRA2a expression, potentially improving cardiac hypertrophy and reducing myocardial fibrosis by inhibiting DNA methyltransferase [164]. Additionally, 5-Aza-2-deoxycytidine (DAC) has been shown to treat these conditions by upregulating the expression of *ERa*, *ERb*, and *COL15A1* in smooth muscle and endothelial cells [165]. Furthermore, 5-Aza-2-deoxycytidine has demonstrated the ability to reverse myocardial proteome changes, decrease myocardial hypertrophy, enhance contractility, and reduce susceptibility to ischemic injury in rats [166]. Acetylsalicylic acid treatment can reduce ABCA1 DNA methylation levels, thereby decreasing the risk of atherosclerosis and coronary heart disease [167]. These findings highlight the potential of DNA methylation inhibitors as therapeutic agents in cardiac conditions.

### 3.2. Histone Deacetylase Inhibitors

HDAC inhibitors (HDACIs) have been demonstrated to effectively regulate both atrial and ventricular fibrosis [168]. *Trichostatin A* (TSA), a pan-HDAC inhibitor, significantly attenuates atrial fibrosis and the associated risk of AF [169]. Additionally, TSA restores normal connexin 40 remodeling, reverses conduction abnormalities, and enhances atrial automaticity. In a canine model of atrial arrhythmia and fibrosis, the administration of both pan-HDACIs and class I HDAC-specific inhibitors successfully mitigated atrial fibrosis and AF. This therapeutic approach also resulted in improved overall cardiac function and a significant reduction in inflammatory cell infiltration [170].

Research has demonstrated that HDACs are crucial in arrhythmia development. Specifically, the deletion of HDAC1 and HDAC2 in mice led to severe cardiac arrhythmia due to the dysregulation of calcium subunits. Additionally, treatment with HDACIs, such as suberanilohydroxamic acid (SAHA), improved calcium handling and contractility in cardiomyocytes, indicating the pivotal role of class I HDACs in atrial fibrosis and arrhythmia [168,171]. HDACIs effectively control fibrosis in both right and left ventricles. Studies show that HDACIs reduce right ventricular fibrosis in models of hypertrophy, unlike ACE inhibitors, but more research is needed in this area. Conversely, the impact of HDACIs on left ventricular hypertrophy and fibrosis shows that these inhibitors alleviate hypertrophy, slow fibrosis progression, and improve HF outcomes. HDACIs are particularly beneficial for diastolic HF with cardiac fibrosis. However, it is unclear if their anti-fibrotic effects are direct or due to improvements in hypertrophy. Research suggests that HDACIs may directly regulate fibrosis by inhibiting myofibroblast differentiation [168,172].

### 3.3. Histone Methyltransferase Inhibitors

Histone methyltransferases (HMTs) are enzymes responsible for adding one to three methyl groups to lysine residues on proteins during post-translational modification [173]. Chaetocin, a histone H3K9 methyltransferase inhibitor, plays a crucial role in preserving chromatin structure by reversing excessive heterochromatinization and mitigating myocardial hypertrophy through the inhibition of H3K9 methyltransferase, making it a promising candidate for future chronic HF therapies [174]. Similarly, Tanshinone IIA, a key active compound of Danshen, has shown significant involvement in cardiovascular disease by reducing H3K9 trimethylation through JMJD2A inhibition. This action leads to the epigenetic silencing of pro-hypertrophic genes, thereby inhibiting maladaptive cardiac remodeling. Additionally, Tanshinone IIA promotes the expression of Nrf2 by facilitating the hypomethylation of the Nrf2 promoter and inhibiting the activity of HDACs [175]. Together, these findings suggest that both chaetocin and Tanshinone IIA have substantial therapeutic potential in managing CVDs. Furthermore, resveratrol has shown promise in treating DOCA salt-induced hypertension by modulating vascular H3K27me3 methylation [162,176].

### 3.4. Non-Coding RNA Modulators

In recent years, a substantial body of evidence has underscored the pivotal role of non-coding RNAs in gene regulation and the pathogenesis of CVDs [177]. Due to their important regulatory functions, non-coding RNAs have emerged as promising targets for potential clinical interventions.

Recent studies have shown that miR-92 plays a beneficial role in modulating transcriptional networks that regulate angiogenesis, cardiac fibrosis, hypertrophy, ECM remodeling, and myocyte growth. MRG-110, a miR-92a antagonist, is currently undergoing phase II clinical trials in patients with ischemic cardiomyopathy and HF [178]. Additionally, a recent study has reported promising results for a new antagomir targeting miR-132. Since miR-132 activation is linked to cardiac remodeling and hypertrophy, inhibiting it with CDR132L, a synthetic oligonucleotide inhibitor, has proven both effective and safe in patients with ischemic HF [179]. Further, the inhibition of miRNA-33a and miRNA-33b has been demonstrated to reduce plasma LDL-C levels while simultaneously increasing plasma HDL cholesterol levels, all without notable adverse effects. This suggests that targeting miRNA-33 could be an important strategy in treating hyperlipidemia [180].

It has been reported that in patients with MI, lncRNA MIAT plays a role in the regulation of Wnt7b by targeting miRNA-150-5p and *VEGF* signaling pathways. This lncRNA is also differentially expressed in the peripheral blood of these patients, suggesting that lncRNA MIAT could serve as a potential therapeutic target and strategy for treating MI [181]. Studies have shown that lncRNA SNHG12 is highly expressed in vascular endothelium but decreases as atherosclerosis progresses. In both pig and human atherosclerotic tissues, lower SNHG12 levels were linked to increased DNA damage and signs of aging. DNA-dependent protein kinase (DNA-PK), essential for DNA repair, interacts with Ku70 and Ku80, but this interaction is disrupted when SNHG12 is reduced, leading to more DNA damage. Injecting SNHG12 has been found to help prevent atherosclerosis by protecting blood vessels from DNA damage and slowing endothelial aging [182]. Furthermore, lncRNAs such as MALAT1, MANTIS, MEG3, and STEEL play a crucial role in regulating endothelial function by influencing apoptosis, cell proliferation, migration, and angiogenesis [182,183,184].

## 4. Conclusions and Future Prospective

CFs play a crucial role in the development of heart fibrosis [185]. Normally, fibroblasts remain dormant and are protected by a stable matrix network in healthy hearts. However, when the heart experiences injury and inflammation, the matrix structure is disrupted, exposing fibroblasts to mechanical stress and growth factor stimulation [186]. Various factors, such as damage-associated molecular patterns (DAMPs), fibroblast growth factor-2, angiotensin II, platelet-derived growth factor, and proteases from mast cells, can activate fibroblasts, leading to their proliferation [187]. Recent advancements in the identification of specific fibroblast lineages have improved our understanding of the cellular origins of CFs. Recent findings challenge previous assumptions that bone marrow-derived and endothelial cells contribute significantly to the population of activated fibroblasts [188]. Resident CFs have emerged as the primary source of fibroblast activation. Epigenetic modifications, including DNA methylation, histone modifications, and non-coding RNAs, have been identified as critical regulators of CF activation [189]. Therefore, targeting these epigenetic mechanisms may offer promising therapeutic strategies for preventing and treating myocardial fibrosis. Further studies are needed to unravel the intricate interplay between different epigenetic modifications and their functional consequences in CF activation, with the ultimate goal of improving patient outcomes in CVDs. This review summarizes the recent advancements in our understanding of the relationship between epigenetic regulation and CFs. Epigenetic modifications play a crucial role in transmitting upstream signals and reprogramming gene transcription, thereby mediating cardiac fibrogenesis [190]. These modifications involve key nodes, including epigenetic-modifying enzymes, which offer potential targets for preclinical studies and the development of clinical protocols to address cardiac fibrosis. The role of epigenetics in the underlying mechanism of fibrosis is important, and it is equally critical to investigate anti-fibrotic factors, particularly those involved in cardiac myofibroblast deactivation. Although limited research currently exists on the relationship between epigenetics and myofibroblast deactivation, future studies in this area are promising because of the reversible nature of epigenetic modifications. An example of a negative regulator of TGFβ1/Smad signaling, SKI, demonstrates potent fibrosis inhibition and the ability to deactivate activated fibroblasts [191]. Examining the regulatory mechanisms underlying myofibroblast deactivation, particularly in the context of epigenetic regulation, may pave the way for novel treatment approaches for cardiac fibrosis. It is important to acknowledge that the regulatory processes of epigenetic modulators are extensive and complex. There is a significant interplay between the different types of epigenetic modifications and their capacity to interact and influence each other. Moreover, intricate feedback regulatory loops further complicate the drawing of generalized conclusions regarding homogeneous epigenetic regulation. Advances in this field will help elucidate the intricacies of epigenetic regulation in the context of cardiac fibroblasts and cardiac dysfunction. In the future, the widespread use of histological assays will help to elucidate the complexity of epigenetic regulation in the context of cardiac fibroblasts and cardiac dysfunction.

## Figures and Tables

**Table 1:** Role of DNA methylation and histone modification in CF activation and cardiac fibrosis.

Epigenetic Modification	Epigenetic Modifiers	Fibrosis Model	Targets	Cardiac Function	References
DNA methylation	DNMT1	Diabetic cardiomyopathy; thoracic aortic constriction; isoproterenol	SOCS3, microRNA- 152-3p	Pro-fibrotic activation, CFs autophagy, CF proliferation	[27,31]
	DNMT3a	Thoracic aortic constriction	TRAAK/RASSF1A, Ras/ERK1/2/miR-200b	Pro-fibrotic, CF activation and proliferation, CF activation/pro-fibrotic	[30,31,34]
	DNMT3b	Hypoxia; thoracic aortic constriction	HIF-1α/Rasal1, Rassf1	Pro-fibrotic, CF activation/pro-fibrotic, CF activation	[33,34]
DNA demethylation	TET2	Angiotensin II; TET2 KO	IL-6, Rasal1,	anti-fibrotic, inflammatory response, anti-fibrotic, protection of cardiomyocyte	[39,41]
	TET3	Thoracic aortic constriction,	Hspa1b	Anti-fibrotic, EndMT	[43]
Histone acetylation	p300	High glucose; angiotensin II; Thoracic aortic constriction	Smad2, H3K9, GATA4	Pro-fibrotic, collagen production/ Pro-fibrotic, CF activation and type I collagen synthesis/ Pro-fibrotic, collagen production	[43,44,45,46,47,48,49,50,51,52,53,54,55,56,57,58,59]
	PCAF (p300/CBP-associated factor)	Isoproterenol-induce rat fibrotic model	Smad2	Pro-fibrotic, CF activation	[55]
Histone deacetylation	HDAC1	Myocardial infarction; thoracic aortic constriction; angiotensin II	Clca2, p53	Pro-fibrotic, CF activation and proliferation	[60,61]
	HDAC2	Isoproterenol	PPP2CA, α-SMA	Pro-fibrotic, α-SMA synthesis/pro-fibrotic, CF activation	[28,62]
	HDAC3	Diabetic cardiomyopathy;	DUSP5	Pro-fibrotic, fibrosis markers and collagen accumulation	[29]
	HDAC8	isoproterenol-induce fibrotic model;	P38 MAPK	Pro-fibrotic, markers of fibrosis	[63]
	BRD4	thoracic aortic constriction	Sertad4/Meox1	Pro-fibrotic, CF activation and proliferation	[64,65]
Histone methylation	EZH1/2,	angiotensin II	PPAR-γ	Pro-fibrotic, Col1a1 and Col3a1 synthesis	[66]
	EZH2	High-Fat; angiotensin II; diabetic cardiomyopathy; thoracic aortic constriction	H3K27me2/3, ACTA2, lncRNA-ANRIL, Smad7	Anti-fibrotic, suppression of pro-fibrotic genes/pro-fibrotic, CF activation and migration/pro-fibrotic, increased expression of FN, Col1α4/pro-fibrotic, CF activation	[67,68,69]
	SUV39H1	SHRs	MyoD	Pro-fibrotic, CF proliferation and collagen accumulation	[70]
	DOT1L,	Myocardial infarction	SYK	Pro-fibrotic, CF activation	[71]
Histone demethylation	LSD1,	Thoracic aortic constriction	TGF-β	Pro-fibrotic, CF activation and collagen secretion	[72]
	KDM3A	Thoracic aortic constriction	Timp1	Pro-fibrotic, CF activation	[73]
	KDM3C	Angiotensin II	Timp1	Pro-fibrotic, CF activation	[74]
	KDM6B	Angiotensin II	β-catenin	Pro-fibrotic, ECM deposition	[75]

**Table 2:** The roles of non-coding RNA modification in CF activation and cardiac fibrosis.

Epigenetic Modification	Epigenetic Modifiers	Fibrosis Model	Targets	Cardiac Function	References
miRNA	miR-23b	Myocardial infarction; angiotensin II; thoracic aortic constriction	FBW7, p53, SPRY3, FGF1, ZEB1, SMAD2, TGIF1, PTEN	Pro-fibrotic, CF proliferation and collagen production	[117]
	miR-27b	Myocardial infarction	FBW	Pro-fibrotic, CF proliferation and collagen production	[115]
	miR-125b	Angiotensin II	Apelin, p53	Pro-fibrotic, CF proliferation	[114]
	miR-99b-3p	Angiotensin II	GSK-3β	Pro-fibrotic, CF proliferation and migration	[113]
	miR-143-3p	Myocardial infarction	SPRY3	Pro-fibrotic, CF activation, proliferation, and migration	[112]
	miR-21	Thoracic aortic constriction	Sprouty1/2 (Spry1/2)	Pro-fibrotic, CF activation	[118]
	miR-27b-3p	Thoracic aortic constriction; angiotensin II	Fgf1	Pro-fibrotic, mitochondrial oxidative phosphorylation	[119]
	miR-9- 5p	Myocardial infarction	Follistatin-like 1 (FSL1)	Attenuated fibrosis and inflammatory response	[120]
	miR-33	Transverse aortic constriction	ABCA1	Cardiac fibrosis, transverse aortic constriction	[121]
	miR- 34a	Myocardial infarction	Smad4	Cardiac fibrosis progression	[123]
	miR-93	Myocardial fibrosis	c-Ski	Myocardial fibrosis	[124]
	miR-130a	AngII-infused model	PPARγ	Cardiac fibrosis, myofibroblasts differentiation	[125]
	miR-144-3p	Myocardial infarction	PTEN	Promotes cell proliferation, migration, and collagen production	[126]
	miR-150-5p	Myocardial fibrosis	EGR1	Myocardial fibrosis	[127]
	miR-221/222	Angiotensin II	SMAD2	Anti-fibrotic, CF activation, and proliferation	[128]
	miR-1954	Angiotensin II	THBS1	Anti-fibrotic, attenuation inflammation	[129]
	miR-590-3p	Myocardial infarction	ZEB1	Anti-fibrotic, CF activation, proliferation, and migration	[130]
	miR-15a/b	Diabetic and non-diabetic patients undergoing coronary artery bypass graft surgery	CTFG	Diastolic dysfunction, fibrosis	[131]
	miR-26b	Myocardial infarction	Smad2/3	Inflammatory reaction, myocardial injury, fibrosis and myocardial cell apoptosis	[132]
	miR-29	Smad 3^+/+^, Smad 3^−/−^, Smad 7^+/+^, Smad 7^−/−^	FBN-1, MMP	Myogenic differentiation, transdifferentiation of myoblasts into myofibroblasts	[133,134]
	miR-101	Myocardial infarction	RUNX1	Myocardial fibrosis, cardiomyocyte apoptosis	[135]
lncRNA	lncRNA AK081284	Diabetic cardiomyopathy	IL-17	Pro-fibrotic, CF proliferation, and collagen production	[136]
	lncRNA AK137033	Myocardial infarction	Sfrp2	Pro-fibrotic, CF activation, and proliferation	[137]
	lncRNA AK048087	Myocardial infarction/ Angiotensin II	COTL1	Pro-fibrotic, CF activation, and proliferation	[138]
	lnc GASS	Isoproterenol	MiR-21	Anti-fibrotic, CF proliferation	[139]
	lncRNA Crnde	Diabetic cardiomyopathy	Smad3	Anti-fibrotic, CF activation	[140]
	lncRNA MANTIS	Myocardial infarction	Sox18, Smad3/6	Anti-fibrotic, vascular neogenesis	[141]
	lnc Neat1	Myocardial infarction	Neat1, P53 HIF2A	Fibroblast and cardiomyocyte survival and functions	[142]
	lnc PCFL	Myocardial infarction	miR-378	Pro-fibrotic, CF proliferation, and collagen production	[143]
	lnc Kcnq1ot1	Streptozotocin (STZ)-induced diabetic (C57BL/6 mice)	Kcnq1ot1, miR-214-3p, caspase-1, TGF-β1	Ameliorated pyroptosis and fibrosis	[144]
	lnc Wisper	Thoracic aortic constriction	TIA1-related protein	Pro-fibrotic, CF proliferation	[145]
	lncMeg3	Thoracic aortic constriction	MMP-2	Pro-fibrotic, ECM deposition	[146]
RNA modification (m^6^A)	METTL3	Myocardial infarction	MetBil, Fibrosis-related genes	Pro-fibrotic, CF activation, and proliferation	[147]
	METTL3	ISO-induced cardiac fibrosis	IGFBP3	Promote cell activation, migration	[148]
	WTAP/YTHDF2	Cardiac fibrosis	Decr1	Diabetic cardiac fibrosis	[149]
	FTO	Myocardial infarction; diabetic cardiomyopathy	Serca2a/CD36, Slc5a33	Anti-fibrotic, CF activation, proliferation, and migration/Anti-fibrotic, collagen deposition suppression	[148]
	ALKBH5	Myocardial infarction	ErbB4	Fibroblast activation	[150,151,152]
